# Custom Three-Dimensional Printed Scaffolds or Implants in Patients With Segmental Bone Loss of the Foot and Ankle: A Single-Centre Case Series

**DOI:** 10.7759/cureus.78309

**Published:** 2025-01-31

**Authors:** Jesse Wells, Andrew Walls, Joel Morash

**Affiliations:** 1 Medicine, Dalhousie University, Halifax, CAN; 2 Trauma and Orthopaedics, The Western Trust Health and Social Care in Northern Ireland (HSCNI), Londonderry, GBR; 3 Orthopaedic Surgery, Dalhousie University, Halifax, CAN

**Keywords:** 3d printed implant, additive manufacturing, ankle, critical defect, custom cage, foot, implant, patient-specific implant, segmental bone loss, titanium

## Abstract

Background: Custom three-dimensional (3D)-printed implants are a novel surgical treatment for a subset of patients requiring foot and ankle surgery for segmental bone loss. Limited outcome data exist in the literature due to the limited number of cases and short follow-up. The objective of this case series was to evaluate the survival of implants, bony union rates, and measures of pain and quality of life in patients who received custom 3D-printed implants for critical defects of the foot and ankle.

Methods: This is a retrospective case series to assess surgical outcomes of patients who underwent implantation of a custom 3D-printed titanium implant between November 2017 and December 2022 in a single foot and ankle unit. The primary outcome was device failure, defined as the removal of the implant. Radiographic analysis was performed to assess for bony integration of implants. Patients completed Short Form 36 (SF-36) and Ankle Osteoarthritis Scale (AOS) questionnaires postoperatively.

Results: Ten consecutive patients, five non-weight bearing on average 10 months (range: eight to 13 months) preoperatively and five weight bearing, underwent surgery with custom implants. The average follow-up was 25 months. To date, no patient has required hardware removal or progressed to amputation. Four patients had radiographic bony union confirmed on CT and one on plain film. Two cases are too early to determine, two are total talus implants with no integration surface, and one has a painless fibrous union. The average SF-36 mental component score was 37.80 (range: 8.1-60.64), and the average physical component score was 32.56 (range: 15.5-54.77).

Conclusion: This case series adds to the growing body of evidence indicating the clinical utility of 3D-printed implants for use in segmental defects of the foot and ankle, demonstrated by the survivorship of implants, promising bony union rates, and functional outcomes. Due to the costs of these implants, larger sample sizes and longer follow-ups are required to support their use.

## Introduction

Critically sized defects (CSDs) are bone losses that exceed the body’s ability to heal. These defects can be defined as bone loss >1-2 cm in length or 50% of the circumference of the bone [1]. Common aetiologies of CSDs include high-energy trauma, infection requiring debridement, tumour resection, and removal of failed total ankle arthroplasty [1,2].

Traditional surgical options for these patients include bone transport with the use of external fixators, bulk allografts, and the induced membrane technique [1,3]. Challenging aspects of these traditional techniques include nonunion, infection, fracture, long-term external fixation, and the requirement of multiple surgeries [4-5]. As a result of a failed intervention, a substantial proportion of patients will have chronically painful extremities and/or require amputation [6].

A novel solution is the use of custom, three-dimensional (3D)-printed porous titanium implants. The advantages of these implants over the current options include the ability to provide patient-matched size and shape needed to fill the defect, thus reducing intraoperative modifications; improved structural integrity compared to allograft bone; lack of invasive harvesting of a patient’s own tissues; and a single operation [3,7-9]. The 3D-printed lattice allows for bone ingrowth that is typically unattainable with traditional manufacturing processes [8]. During the manufacturing process, the void space can be modified to change mechanical properties such as stiffness and elasticity in order to meet the needs of the anatomical area [10-11]. This allows for improved integration of the bony surface into the implant with the hope of improving long-term stability and reducing post-operative failure.

Due to the novelty of the therapy, limited use, and short follow-up time, the long-term outcomes in patients who have 3D-printed custom implants are limited, and the literature characterising their efficacy is still in its infancy [3,12-14]. Understanding union rates and patient-reported and functional outcomes of individuals who receive these implants is critical to establishing their utility in these high-risk patients. The purpose of this case series is to evaluate and report the failure rate as well as initial functional and patient-reported outcomes in consecutive patients receiving custom 3D-printed titanium implants to treat CSD of the foot and ankle.

## Materials and methods

Study design

This is a retrospective case series to assess the surgical outcomes of patients who underwent implantation with a custom 3D-printed titanium implant between November 2017 and December 2022 in a single foot and ankle unit. Consecutive patients with CSDs consented to the intervention after a discussion detailing the risks and benefits of all surgical options. All surgeries were performed by a single surgeon at the Queen Elizabeth II Health Sciences Centre (QEII) in Halifax, Canada.

A case review of the notes was conducted assessing the survivorship of the implants and complications in the operative course. The primary outcome was device failure, defined as the removal of the implant. Indications for removal of the implant included septic or aseptic nonunion. Secondary outcomes included patient-reported outcomes, assessed by Short-Form-36 (SF-36) [15] and Ankle Osteoarthritis Scale (AOS) [16] completed post-operatively, functional outcomes, and bony union assessed radiographically. Statistical analysis consisted of descriptive statistics, including mean and standard deviation of SF-36 and AOS scores.

Demographic and comorbidity data were collected, including diabetes status, Charlson Comorbidity Index (CCI) [17], and smoking history. Surgical information collected included the duration of surgery, bone grafts/substances used, and post-operative complications. Radiographic analysis was completed to assess for bony integration of implants via plain film X-ray and CT when required. All patients were followed up in normal protocol unless there were wound concerns. The first review was at two to three weeks postoperatively, then six weeks, three months, six months, and a year, respectively. A CT was requested if there were concerns about no bony integration with the cage construct and/or if the patient had significant pain. The discharge time point was case-specific. The majority of cases were kept under long-term review on an annual basis due to the nature of the implants.

Three-dimensional implant design

**Figure 1:**
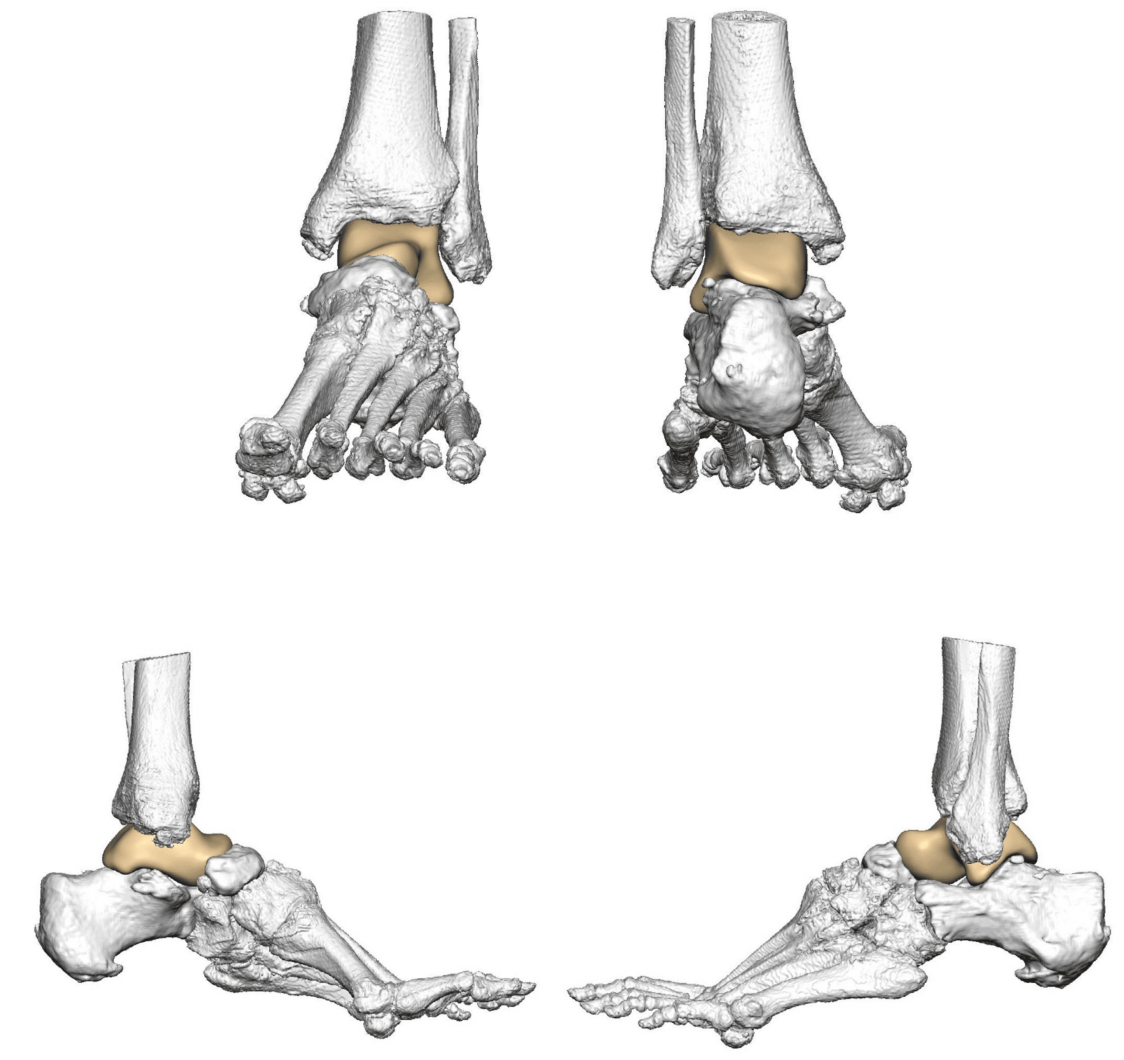
4WEB MedicalTM design proposal of a total talus replacement based on CT imaging of a patient’s extremity Figure published with permission from 4WEB Medical^TM^ [18]

**Figure 2:**
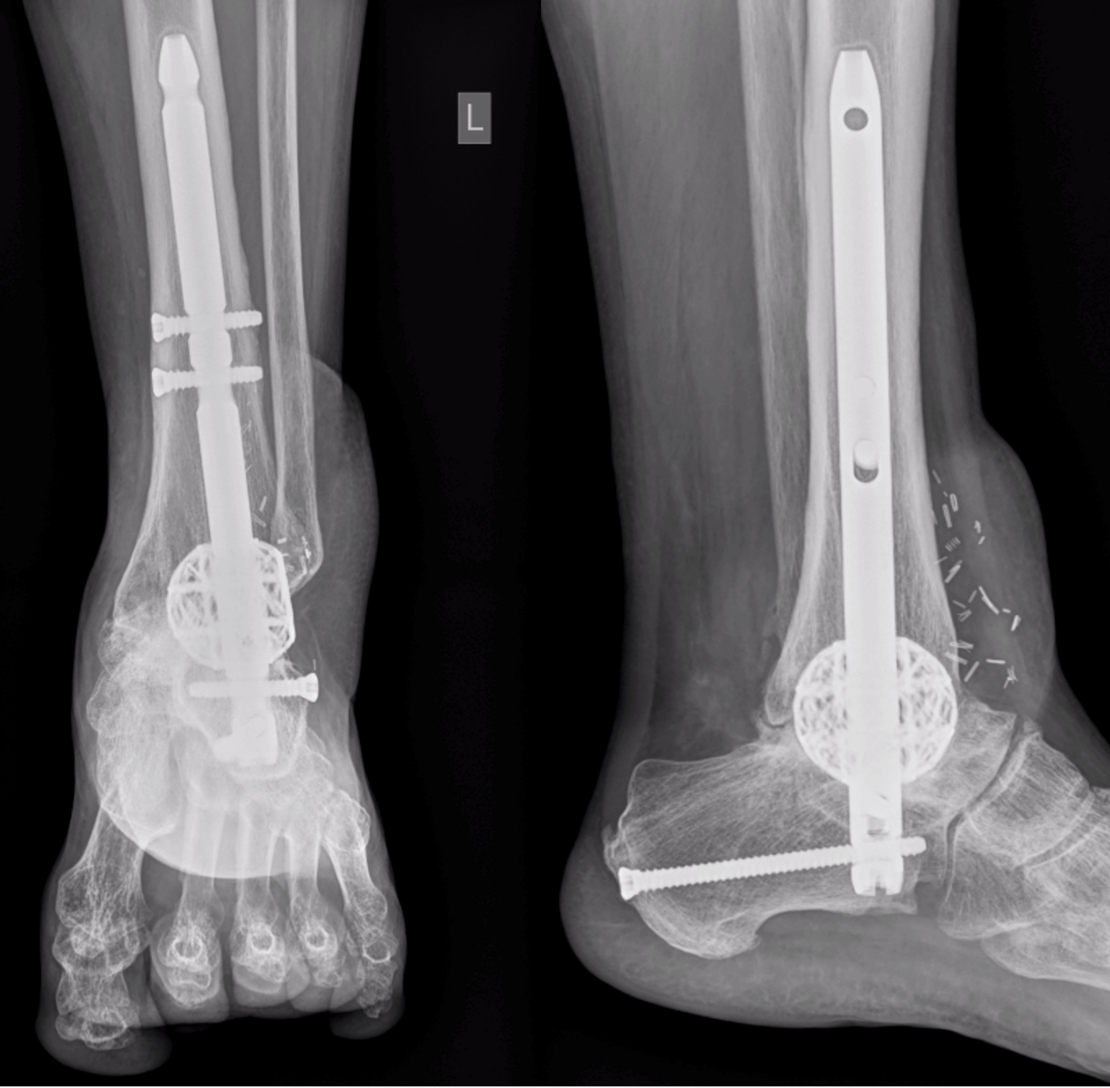
Anterior-posterior (left) and lateral (right) X-rays of a successful implant that did not require revision surgery. The patient is a 56-year-old polytrauma patient who underwent a custom 3D-printed cage with a tibiotalocalcaneal nail following washout and cement spacer insert.

Each implant was custom-engineered by 4WEB Medical^TM^ (Frisco, TX) using proprietary technology and CT imaging of patient extremities [18]. The process involves CT scans of the patient's involved extremity being sent to the device manufacturer. Subsequently, a team of engineers works with the surgeon to design a 3D-printed implant specific to the patient's defect. After agreement on the design, the implant is printed, processed, and sent to the hospital for sterilisation. The implant is engineered with hierarchical surface roughness to facilitate bony growth and fusion. In addition, the open architecture and cage design allow for the even distribution of weight to reduce the risk of subsidence. Figure 1 below contains an example of a design proposal for a total talus replacement in this series. Figure 2 displays post-operative plain film X-rays of a custom 3D-printed cage with a tibiotalocalcaneal nail for a post-traumatic critically sized defect.

Ethical approval

Ethical approval was obtained from the Nova Scotia Health Research Ethics Board, Halifax, Canada, under file number 1020336.

## Results

Ten consecutive patients during the case review period of November 2017 to December 2022 underwent hindfoot surgery with custom 3D implants for total talus replacement or ankle arthrodesis. The indications for implantation included post-traumatic hindfoot deformity (n=7), spontaneous talar body avascular necrosis (n=2), and Charcot ankle deformity (n=1). 

Demographic data of the study group are outlined in Table 1. Five patients were non-weight bearing, on average 10 months (range: eight to 13 months) prior to surgery. The remaining five were weight bearing prior to surgery. The average age at surgery was 54.5 years (range: 17 to 76 years). The average age at follow-up was 56.5 years (range: 22 to 76 years). The average follow-up time in months was 25.1 months (range: two to 63 months). Nine patients were non-diabetic, and one had type II diabetes. The average CCI was 1.8 (range: 0 to four), leading to an average estimated 10-year survival rate between 90% and 96%. Five patients had never smoked tobacco, five were ex-smokers, and two were cannabis smokers. The average duration of surgery, defined by the time of first incision to time of closure, was 2.5 hours (range: 1.5 to four hours). Upon intraoperative decision-making by the operating surgeon, one patient had an autograft only, six patients had an autograft with chips and a synthetic graft (MATRix4, Memphis, TN), one patient had an autograft with a bone void filler, and two patients did not have a graft. 

**Table 1:** Demographic data of patients who underwent the implant of a 3D-custom cage for hindfoot deformity

Indication	Number (n)	Range
Post-traumatic hindfoot deformity, n	7	
Spontaneous talar body avascular necrosis, n	2	
Charcot ankle deformity, n	1	
Sex		
Male, n	6	
Female, n	4	
Age at surgery, years, mean	54.5	17 – 76
Age at follow-up, years, mean	56.5	22 – 76
Follow-up time, months, mean weight bearing status	25.1	4 – 63
Non-weight bearing prior to surgery, n	5	
Weight bearing prior to surgery, n	5	
Diabetes status		
Non-diabetic, n	9	
Diabetic, n	1	
Charlson Comorbidity Index, mean	1.8	0 – 4
Smoking status		
Non-smoker, n	4	
Previous smoker, n	5	
Current cannabis smoker, n	2	
Duration of surgery, hours, mean	2.5	1.5 – 4

Table 2 outlines the post-operative measurements. To date, all of the implants have survived with no need for explant and no amputations. In terms of complications, one patient had post-operative paraesthesia in the sural nerve distribution, and one patient had wound dehiscence requiring debridement, which was successfully completed. The other eight patients had no reported complications. Four patients had radiographic bony union confirmed on CT and one on plain film. Two cases are too early to determine, two are total talus implants with no integration surface, and one has a painless fibrous union. The average mental component score post-operatively was 37.80 (range: 8.1-60.64, SD 16.58) assessed at a minimum of six months post-operatively. The average physical component score was 32.56 (range: 15.45-54.77, SD 12.28) post-operatively. The AOS score can be divided into pain and disability components. The average pain score was 34.44 (range: 12-60, SD 18.47), and the average disability score was 58 (range: 29-74, SD 14.33) post-operatively.

**Table 2:** Outcome measures, including union rates and mean and SD of patient questionnaire scores of patients who underwent the implant of a 3D-custom cage for hindfoot deformity.

Failure of implant to date	Number (n)	Standard deviation (SD)	
Yes, n	0		
No, n	10		
Bony union			
Yes, CT confirmed, n	4		
Yes, plain film, n	1		
Fibrous union, n	1		
No, n	0		
N/A (talar body replacement), n	2		
Too early to determine, n	2		
Short Form 36 (SF-36) Score			
Physical component score, mean	32.56	12.28	
Mental component score, mean	37.80	16.58	
Ankle Osteoarthritis Scale (AOS)			
Pain, mean	34.44	18.47	
Disability, mean	58	14.33	
Total			
Non-diabetic, n	9		
Diabetic, n	1		
Post-operative mobility status			
Weight bearing, n	10		
Non-weight bearing, n	0		

## Discussion

The current literature on total talus replacements shows moderately favourable outcomes with up to 67% improvements in functional outcomes in systematic reviews of current case series [12-13]. In our series, of the eight patients with implants containing an integrative surface, four had union confirmed on CT, one on plain film, and one had what we suspect to be a fibrous union into the implant surface with no failure. Two were too early to determine. Complication rates were 20%. This case series adds to the growing literature demonstrating the clinical utility of custom 3D-printed cages for use in segmented bone defects of the foot and ankle. Of the 10 patients in this series, none have required removal of hardware, demonstrating good survivability over the follow-up period. This adds to the growing evidence of the clinical utility of these implants and their ability to achieve union where the patient may otherwise have gone on to amputation. Similar case series with larger sample sizes and longer follow-up times have demonstrated similar or higher union rates in up to 95% of patients [10,13,19].

Eight patients were fully weight-bearing during follow-up, and one patient was 75% weight-bearing in a walking boot. This demonstrates a good functional outcome relative to more traditional treatments, which would involve larger areas of graft and bridging metalwork and would require longer periods of immobilisation and casting. The structural titanium shells of the custom 3D implants allow for more aggressive and early post-operative mobilisation and rehabilitation.

The average SF-36 scores for the mental component in this case series are approaching the normative value of 50. The physical component of the SF-36 score is below the normative value at an average value of 32.56, suggesting a greater degree of functional impairment compared to psychological impairment. The average AOS score in this series was higher for disability than for pain. It is difficult to draw significant conclusions from this data given that we did not assess patient-perceived experience on SF-36 and AOS scales pre-operatively, and therefore we do not have any pre-operative comparative values. Future studies should address this gap and may also consider comparing subjects to matched-control subjects who undergo traditional surgical methods for CSD. Patient outcome scores in this series are not optimal or normative, yet the alternative in many of these cases would have been amputation, an option that they did not choose in the consent process.

This series has several limitations, including a small sample size, a wide range of follow-up time, and the lack of pre-operative patient-reported mental and physical scores. Future studies may consider administering patient-reported physical and mental status prior to surgery and at spaced intervals post-operatively in order to compare post-operative to pre-operative scores and assess how scores change over time.

## Conclusions

This case series adds to the growing literature demonstrating the clinical utility of custom 3D-printed cages for use in segmented bone defects of the foot and ankle. This is demonstrated by the survivorship of implants, promising bony union rates and positive functional outcomes. Although the field of 3D-manufactured implants for use in critically sized defects is promising, due to their high cost, larger sample sizes and longer follow-ups are required to support their use.

## References

[1] Nauth A, Schemitsch E, Norris B, Nollin Z, Watson JT (2018). Critical-size bone defects: is there a consensus for diagnosis and treatment?. J Orthop Trauma.

[2] Schemitsch EH (2017). Size matters: defining critical in bone defect size!. J Orthop Trauma.

[3] Abar B, Kwon N, Allen NB, Lau T, Johnson LG, Gall K, Adams SB (2022). Outcomes of surgical reconstruction using custom 3D-printed porous titanium implants for critical-sized bone defects of the foot and ankle. Foot Ankle Int.

[4] Bakri K, Stans AA, Mardini S, Moran SL (2008). Combined massive allograft and intramedullary vascularized fibula transfer: the capanna technique for lower-limb reconstruction. Semin Plast Surg.

[5] Baldwin P, Li DJ, Auston DA, Mir HS, Yoon RS, Koval KJ (2019). Autograft, allograft, and bone graft substitutes: clinical evidence and indications for use in the setting of orthopaedic trauma surgery. J Orthop Trauma.

[6] Saddawi-Konefka D, Kim HM, Chung KC (2008). A systematic review of outcomes and complications of reconstruction and amputation for type IIIB and IIIC fractures of the tibia. Plast Reconstr Surg.

[7] Dekker TJ, Steele JR, Federer AE, Hamid KS, Adams SB Jr (2018). Use of patient-specific 3D-printed titanium implants for complex foot and ankle limb salvage, deformity correction, and arthrodesis procedures. Foot Ankle Int.

[8] Kadakia RJ, Wixted CM, Kelly CN, Hanselman AE, Adams SB (2021). From patient to procedure: the process of creating a custom 3D-printed medical device for foot and ankle pathology. Foot Ankle Spec.

[9] Abar B, Alonso-Calleja A, Kelly A, Kelly C, Gall K, West JL (2021). 3D printing of high-strength, porous, elastomeric structures to promote tissue integration of implants. J Biomed Mater Res A.

[10] Raikin SM, Moncman TG, Raikin J (2022). Improved pain and function after TTC fusion with a custom cage. Foot Ankle Int.

[11] Moghaddam NS, Andani MT, Amerinatanzi A (2016). Metals for bone implants: safety, design, and efficacy. Biomanuf Rev.

[12] Bischoff A, Stone R, Dao T, Anderson S, Hill Z, Steginsky B, Mendicino R (2023). Functional outcomes and complications associated with total talus arthroplasty: a systematic review. Foot Ankle Spec.

[13] Jennison T, Dalgleish J, Sharpe I, Davies M, Goldberg A (2023). Total talus replacements. Foot Ankle Orthop.

[14] Steele JR, Cunningham DJ, Adams SB (2019). Comparison of custom 3D printed spherical implants vs femoral head allografts for Tibiotalocalcaneal arthrodeses. Foot Ankle Orthop.

[15] Ware JE, Snow K, Kosiniski M, Gandek B (1993). SF-36 Health Survey Manual and Interpretation Guide. https://www.researchgate.net/profile/John-Ware-6/publication/313050850_SF-36_Health_Survey_Manual_Interpretation_Guide/links/594a5b83aca2723195de5c3d/SF-36-Health-Survey-Manual-Interpretation-Guide.pdf.

[16] Domsic RT, Saltzman CL (1998). Ankle osteoarthritis scale. Foot Ankle Int.

[17] Quan H, Li B, Couris CM (2011). Updating and validating the Charlson comorbidity index and score for risk adjustment in hospital discharge abstracts using data from 6 countries. Am J Epidemiol.

[18] (2024). WEB Medical. https://4webmedical.com/.

[19] Parry E, Catanzariti AR (2021). Use of three-dimensional titanium trusses for arthrodesis procedures in foot and ankle surgery: a retrospective case series. J Foot Ankle Surg.

